# Long Non-coding RNA DUXAP8 Regulates the Cell Proliferation and Invasion of Non-small-cell Lung Cancer

**DOI:** 10.1515/biol-2019-0022

**Published:** 2019-12-31

**Authors:** Si-Jia Yang, Jia-Lu Weng, Bin Wei, Xue-Kui Du

**Affiliations:** 1 Department of Respiratory Medicine, Ningbo No.2 Hospital, No. 41, Northwestern Street, Ningbo, Zhejiang Province, 315099, China; 2 Department of ophthalmology and otorhinolaryngology, Ningbo No.2 Hospital, No. 41, Northwestern Street, Ningbo, Zhejiang Province, 315099, China; 3 Department of pulmonology, Ningbo No.2 Hospital, No. 41, Northwestern Street, Ningbo, Zhejiang Province, 315099, China

**Keywords:** Non-small-cell lung cancer (NSCLC), long non-coding RNA DUXAP8, proliferation and invasion

## Abstract

To investigate how long non-coding RNAs DUXAP8 (LncRNA DUXAP8) influence the cell proliferation and invasion of non-small-cell lung cancer (NSCLC), we detected the expression levels of LncRNA DUXAP8 in lung cancer (LC) tissues, 4 LC-related cell lines (A549, SPC-A1, SK-MES-1 and NCI-H1299) and normal lung tissues via quantitative real-time PCR (qRT-PCR). Compared with normal lung tissue, LncRNA DUXAP8 was significantly up-regulated in NSCLC, especially in stage III / IV and diameter ≥ 3cm of lung cancer. Among 4 lung cancer cell lines, LncRNA DUXAP8 in A549 cells was the highest (*P*<0.001). Construction of LncRNA DUXAP8 overexpression and LncRNA DUXAP8 knockout in A549 cell lines was further performed and subsequently injected into nude mice to build an in vivo tumor xenograft model. The results indicated that LncRNA DUXAP8 overexpression significantly promoted the A549 cells’ proliferation, enhanced invasion and induced tumor growth. Conversely, LncRNA DUXAP8 knockout significantly suppressed A549 cells’ proliferation, weakened invasion and inhibited tumor growth. Taken together, our results imply that LncRNA DUXAP8 is a potential regulatory molecular marker in non-small-cell lung cancer.

## 1 Introduction

Lung cancer (LC) is a common and highly fatal respiratory disease; among them, non-small-cell lung cancer (NSCLC) accounts for more than 85% of all lung cancer cases [1] and its morbidity and mortality ranks the first of all tumor diseases [2]. Surgery, radiation therapy, chemotherapy, and combined modality therapy are common approaches that treat patients with NSCLC. Despite some progress in recent years, patients with NSCLC still have high mortality and poor prognosis.

The participation of long non-coding RNAs (LncRNAs) has a wide repertoire in biological processes of various cancers, such as NSCLC, diffuse large B-cell lymphoma (DLBCL), liver cancer, ovarian cancer, colorectal cancers (CRC), etc. [3, 4, 5, 6, 7, 8]. Recent improvements have revealed that LncRNAs might become a potential biological marker involved in NSCLC pathogenesis and provide new insights into therapy approaches [9, 10]. Accumulating evidence has shown that LncRNAs are involved in regulating the proliferation and invasion of NSCLC [3, 11, 12, 13, 14, 15, 16]. LncRNAs are expected to provide a new target for the research, treatment, and diagnosis of NSCLC.

LncRNAs DUXAP8 is a kind of long non-coding RNA located on chromosome 20q11 with a length of 2307 bp [17]. Several studies suggest that LncRNAs DUXAP8 regulate cell proliferation and migration in several cancers, such as bladder cancer, gastric cancer, and esophageal squamous cell cancer [18, 19, 20]. However, few studies involve LncRNAs DUXAP8 in NSCLC until now. In this study, we examine the expression of LncRNA DUXAP8 in non-small-cell lung cancer and cells and further analyze its relationship to cell proliferation and invasion. Our findings will provide new evidence for the diagnosis and therapy of NSCLC.

## 2 Material and Methods

### 2.1 NSCLC patients and sampling

A total of 43 patients with NSCLC (including 26 males and 17 females; aged 35-75 years) were sampled from Ningbo No. 2 Hospital between January 2016 and November 2017. Among them, 8 cases were in stage I, 14 cases were in stage II, 12 cases were in stage III, 9 cases were in stage IV; 19 cases had tumor diameter ≥ 3cm, and 24 cases had tumor diameter < 3cm; 20 cases were with lymph node metastasis, and 23 cases were without lymph node metastasis; 18 cases were distal metastasis, and 25 cases were non-distal metastasis. All patients were diagnosed with NSCLC based on histopathological features and hematology examination, and categorized into different stages through TNM staging system of malignant tumors after surgery. These patients had not undergone local or systemic treatment before surgery. All 43 healthy volunteers, aged 38–76 years, were selected as the normal group through a routine physical examination. None of them had lung-related diseases or any antibiotics in the past 3 months. All collected tissue samples were immediately snap-frozen in liquid nitrogen and stored at −80°C until required.

**Informed consent:** Informed consent has been obtained from all individuals included in this study.

**Ethical approval:** The research related to human use has been complied with all the relevant national regulations, institutional policies and in accordance the tenets of the Helsinki Declaration, and has been approved by the Ningbo No. 2 Hospital Ethics Committee.

### 2.2 Cell culture

Four human NSCLC cell lines (A549 cells, SPC-A1 cells, SK-MES-1 cells, NCI-H1299 cells) and one normal 16HBE cell line were purchased from Shanghai Institute of Cell Resource Center, CAC, China. 16HBE cells were cultured in RPMI 1640 medium supplemented with 10% fetal bovine serum (FBS) (Sijiqing Biological Material Co., Ltd., Hangzhou, China). A549 cells were cultured in F-12K medium containing 10% FBS. SPC-A1 cells were cultured in DMEM medium containing 10% FBS. SK-MES-1 cells were cultured in EMEM medium containing 10% FBS. NCI-H1299 cells were cultured in RPMI-1640 medium containing 10% FBS. All cells were cultured in humidified air with 5% CO_2_ at 37℃.

### 2.3 RNA extraction, reverse-transcription and qRT-PCR

Total RNA from NSCLC tissue or cells was extracted by Trizol reagent (Invitrogen) according to the manufacturer’s instructions. 2 μg of total RNA was reverse transcribed to cDNA by Reverse Transcription Kit (Takara, Dalian, China). qRT-PCR was performed by using SYBR® Green Master Mix Kit (Takara, China) following the manufacturer’s instructions. The 2^-ΔΔCT^ method was used to calculate the mRNA relative expression level. β-actin was used as the internal standard.

### 2.4 LncRNA DUXAP8 overexpression and knockout treatments

LncRNA DUXAP8 overexpression and knockout were performed using the Lenti-Pac HIV expression packing Kit. 2×10^6^ HEK-293T cells were inoculated into a 10 cm cell culture dish and cultured for 1 day before being subjected to a lentivirus packaging system. 5 μL Lenti-Pac HIV Mix and 2.5 μg plasmid were mixed in 200 μL serum-free medium; then 15 μL EndoFectin Lenti was added, and the mixture was transferred into HEK-293T cells after incubation for 20 min at room temperature, replacing the medium after transfection for 8 h. The medium was added with 20 μL TiterBoost Reagent, and lentivirus was collected after 48 hours. 200 μL of virus solution and 1.5 mL of medium were added into A549 cells. After 48 hours, 0.5 μg/mL puromycin was added to select the stable cell line.

### 2.5 CCK-8 assay

The CCK-8 cell counting kit (Solarbio) was used to determine the cell viability. A549 cells treated with knockout and overexpression were seeded onto 96-well plates and were measured at 24h, 48h and 72h after treatment. In each well, 100 μL CCK-8 solution (10%) was added and incubated for 90 min at 37°C. The optical density at 450 nm was used as the positive index of cell viability by a microplate reader.

### 2.6 Tumor xenograft

A total of 30 BALB/c nude mice were purchased from Zhejiang University Animal Experimental Center. They were divided into two groups: (1) LncRNA DUXAP8 overexpression group (OV, injected with A549 cells with LncRNA DUXAP8 overexpression); (2) LncRNA DUXAP8 knockout group (SI, injected with A549 cells with LncRNA DUXAP8 knockout); (3) Control group (EV, injected with A549 cells as the control). 100 μL of the above cell suspension (1×10^7^ cells) were injected into the right axillary fossa of each mouse through microsyringes. Tumor length (L) and width (W) were measured every 5 days using a digital caliper, and then calculated the tumor volumes (V) following the formula: V = L×W^2^/2. These mice were euthanized and the tumor tissues were weighed after 15 days. The experiments and protocols were approved by the Institutional Animal Care and Use Committee at the Ningbo No. 2 Hospital and all the treatments were performed in accordance with the relevant guidelines and regulations.

### 2.7 Transwell invasion assay

Pre-cooled F-12K medium and Matrigel were mixed in a 1:1 ratio and 0.1 mL was evenly added to the bottom of the upper chamber and incubated for 4 hours. 0.2 mL A549 cells of each group (3×10^4^ cells/well) in the phase of logarithmic growth were added to the upper chamber, and 0.5 mL F-12K medium (10% FBS) was added to the lower chamber. After 48 hours of culture, crystal violet staining was performed, and 5 fields were randomly selected to count.

### 2.8 Statistical analysis

Each treatment was conducted 3 times to obtain the average. All data were expressed as mean ± SD (N=3). The difference comparison was expressed using the SNK-Q test in SPSS v23.0 software. P < 0.05 was considered statistically significant.

## 3 Results

### 3.1 LncRNA DUXAP8 is abundantly expressed in NSCLC

**Figure 1:**
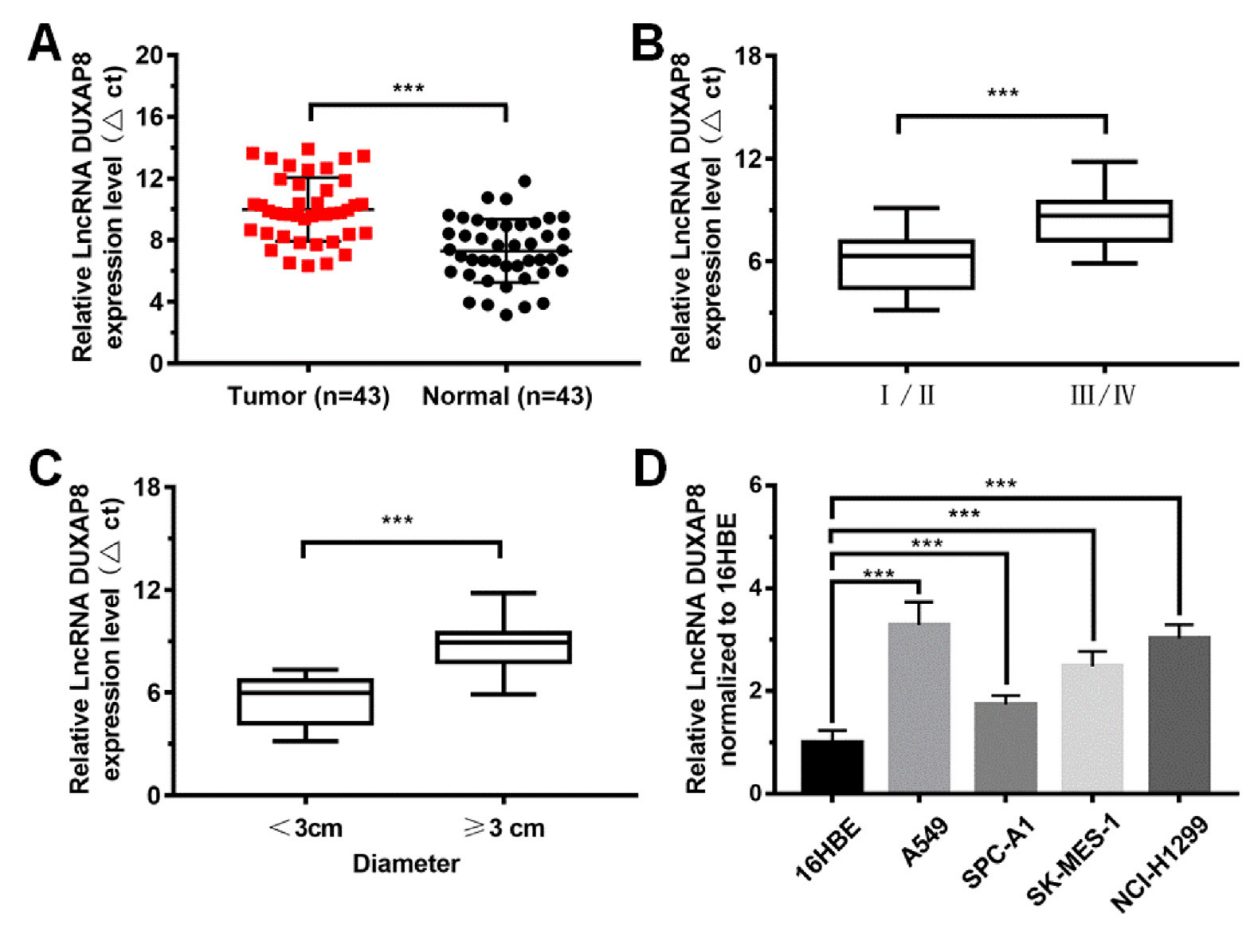
**Expression of LncRNA DUXAP8 in non-small-cell lung cancer tissue and tumor-related cells.** (A) LncRNA DUXAP8 in tumor tissues was significantly higher than that in normal tissue; (B) Expression of LncRNA DUXAP8 in different stages of NSCLC; (C) Expression of LncRNA DUXAP8 in tumor with different diameters; (D) LncRNA DUXAP8 expressed in 4 different lung cancer cell lines. Data were shown as mean ± SD, *** P<0.001.

To determine the relationship between LncRNA DUXAP8 and non-small-cell lung cancer, we examined the expression of LncRNA DUXAP8 in lung cancer tissues and cells (Fig. 1A, 1B and 1C). The expression of LncRNA DUXAP8 in tumor tissues was significantly higher than that of the normal tissue (P<0.001). LncRNA DUXAP8 are differentially expressed in various stages of NSCLC, with higher upregulation in stage III/IV compared to stage I/ II (P<0.001). Meanwhile, the larger the tumor diameter, the higher the LncRNA DUXAP8 expression, with the expression in tumor diameter ≥ 3cm significantly higher than that in tumor diameter < 3 cm (P<0.001). Compared with normal cells (16HBE cells), LncRNA DUXAP8 in A549, SPC-A1, SK-MES-1 and NCI-H1299 were significantly up-regulated (P < 0.001), and the highest expressed in A549 cells (Fig. 1D). These results indicated that LncRNA DUXAP8 may contribute to NSCLC development.

### 3.2 LncRNA DUXAP8 promoted NSCLC cell proliferation

**Figure 2:**
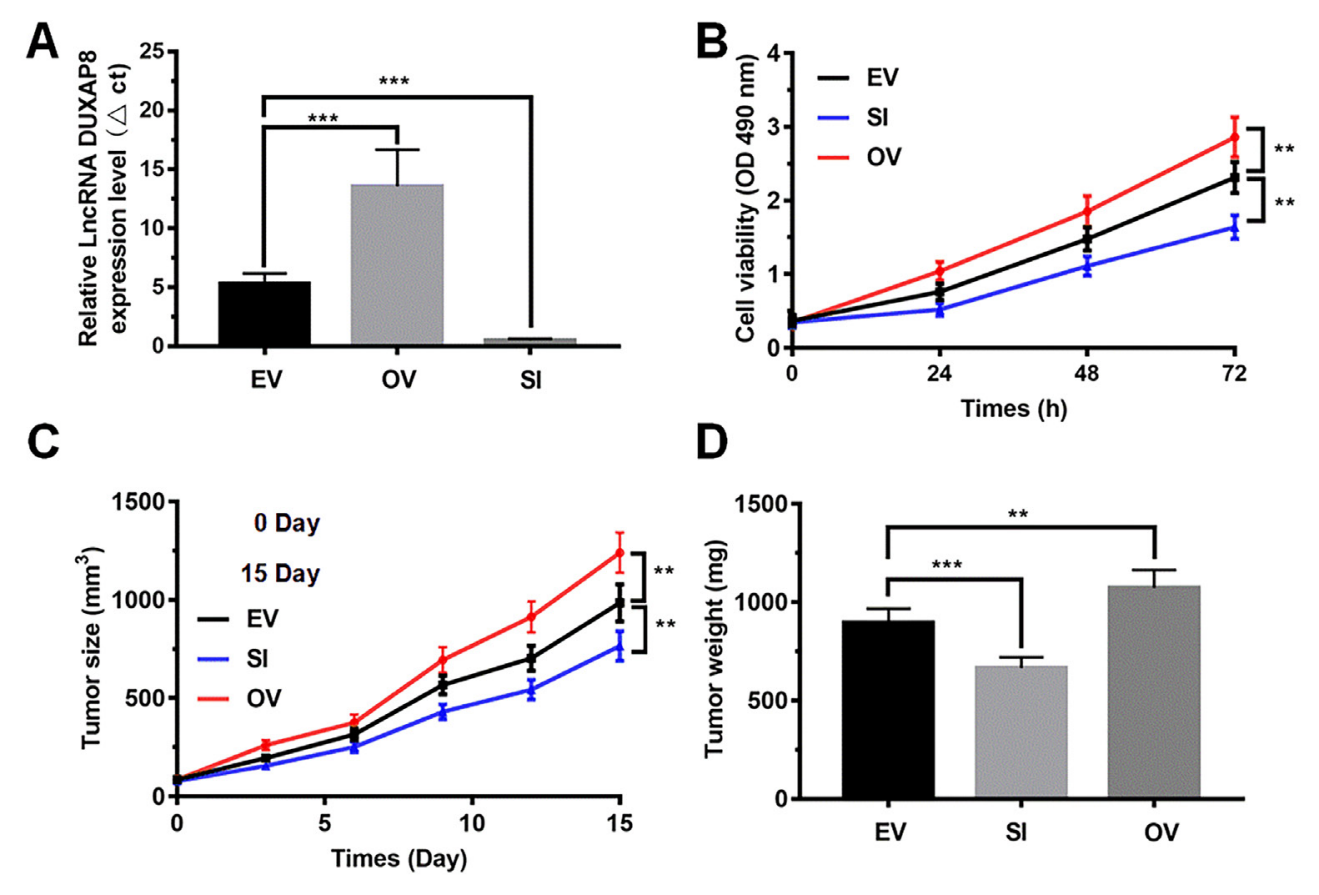
**Effect of LncRNA DUXAP8 overexpression and knockout on A549 cell proliferation.** (A) The efficiency of LncRNA DUXAP8 overexpression and knockout in A549 cells was measured by qRT-PCR; (B) Cell viability in A549 cells was measured by CCK-8 assay at 24h, 48h and 72h after LncRNA DUXAP8 overexpression and knockout; (C) and (D) Tumor volumes and tumor weight were recorded every 5 days. EV: the nude mice were injected with A549 cells; SI: the nude mice were injected with A549 cells with LncRNA DUXAP8 knockout; OV: the nude mice were injected with A549 cells with LncRNA DUXAP8 overexpression. Data are shown as mean ± SD, **P<0.01, *** P<0.001.

To reveal the effect of LncRNA DUXAP8 on NSCLC cell proliferation, LncRNA DUXAP8 overexpression and knockout in A549 cells were modulated (Fig. 2A). The CCK8 assay showed that knockdown of LncRNA DUXAP8 significantly inhibited cell proliferation, whereas LncRNA DUXAP8 overexpression significantly enhanced cell viability (P < 0.001, Fig. 2B). Tumor xenograft was further constructed based on A549 cells with LncRNA DUXAP8 overexpression and knockout (Fig. 2C and 2D). The tumor grew slowly when LncRNA DUXAP8 was knocked out; in contrast, LncRNA DUXAP8 overexpression rapidly promoted tumor growth (considering tumor volumes and tumor weight; compared with EV group, P < 0.001).

### 3.3 LncRNA DUXAP8 facilitated NSCLC cell invasion

**Figure 3:**
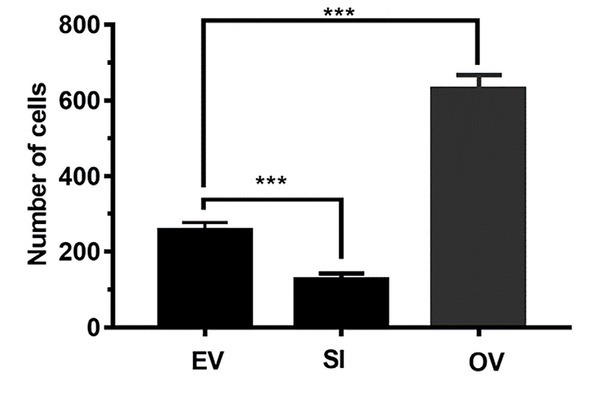
**Transwell invasion assay detected the effect of LncRNA DUXAP8 overexpression and knockout on the invasion of A549 cells.** EV: the nude mice were injected with A549 cells; SI: the nude mice were injected with A549 cells with LncRNA DUXAP8 knockout; OV: the nude mice were injected with A549 cells with LncRNA DUXAP8 overexpression. Data are shown as mean ± SD, *** P<0.001

To assess the effect of LncRNA DUXAP8 on NSCLC cell invasion, transwell invasion assay (Fig. 3) showed that LncRNA DUXAP8 knockout significantly inhibited cell invasion, whereas LncRNA DUXAP8 overexpression promoted cell invasion (P<0.001).

## 4 Discussion

Non-small-cell lung cancer has strong abilities of cell proliferation, invasion and migration [21, 22]. Clinical data suggest that approximately 75% of patients with NSCLC are at a later period of diagnosis, resulting in a lower 5-year survival rate [23]. Non-coding RNAs (ncRNAs) are divided into three classes in terms of the predicted functions: cellular debris ncRNAs (e.g. microRNAs, small nucleolar RNAs), housekeeping ncRNAs (e.g. tRNAs, rRNAs), and regulatory ncRNAs (e.g. lncRNAs and small ncRNAs including microRNAs, small nucleolar RNAs, small interfering RNAs, small nuclear RNAs, and PIWI-interacting RNAs). Theoretical and experimental studies have suggested that ncRNAs play crucial roles in cell proliferation, growth, and migration. LncRNAs are classified into oncogenic lncRNAs and tumor suppressor lncRNAs, and the expression of LncRNAs have been clarified to be associated with NSCLC prognosis.

In this study, qRT-PCR showed that LncRNA DUXAP8 were significantly up-regulated in NSCLC tissues compared with non-tumor lung tissues, especially in stage III / IV and tumor diameter ≥ 3 cm. DUXAP8 is a new oncogenic pseudogene which is significantly enhanced in NSCLC tissues [17]. The expression of LncRNA DUXAP8 was correlated with tumor stage, tumor size, lymphatic metastasis, prognosis, etc. Numerous LncRNAs have different expressive abundance in various tumor stages and tumor sizes of NSCLC[16, 24, 25], such as LncRNAs RNA NEAT1, LncRNAs ANRIL, LncRNAs RNA TUG1, etc. Although it is necessary to further demonstrate the endogenous force in NSCLC patients through LncRNA DUXAP8 expression and pathological data, the differential expression levels of LncRNA DUXAP8 in different stages and sizes of tumors provide an opportunity to diagnose and predict the stages and progress of tumors.

Traditional qRT-PCR has been applied to detect the expression profile of lncRNAs and microRNAs. However, in order to achieve the various requests for clinical applications, faster and more accurate amplification detection methods, such as RT-free qPCR, PCR-based arrays and next generation sequencing, need to be designed and developed considering the accuracy and efficiency of extraction and detection methods.

When LncRNA DUXAP8 was knocked out, in this study, A549 cells’ viability was significantly suppressed. However, the overexpression of LncRNA DUXAP8 can promote NSCLC-related cell proliferation and invasion. This result is similar to Sun *et al*. [17], which suggests that LncRNA DUXAP8 may be an important regulator of cell proliferation and migration in NSCLC. The potential mechanisms by which LncRNAs participate in the regulation of NSCLC processes have been described: (1) via interacting with miRNA, for example, long non-coding RNA NEAT1 promotes NSCLC progression via miR-377-3p-E2F3 axis pathway [24]; (2) via regulating some key genes, for example, LncRNAs DUXAP8 promotes gastric cancer cell proliferation and migration through epigenetically silencing PLEKHO1 (pleckstrin homology domain containing O1) expression [18], whereas it promotes proliferation of bladder cancer cells by regulating the PTEN gene, which had the capacity to inhibit cell growth[19]; (3) via binding some crucial domain, for example, LncRNA DUXAP8 could directly bind with histone methyltransferase EZH2 (a key catalytic subunit of PRC2) and histone demethylase LSD1, implying that LncRNA DUXAP8 regulates gene transcription through epigenetic modification [17]. Our findings indicate that knockdown or overexpression of LncRNA DUXAP8 directly influenced the A549 cells’ viability, which further implied LncRNA DUXAP8 could regulate cell proliferation and migration and become a potential disease biomarker for the screening and early diagnosis of NSCLC.

The tumor xenograft models *in vivo* were constructed by injecting A549 cells with LncRNA DUXAP8 into nude mice. LncRNA DUXAP8 overexpression induced tumor growth, but LncRNA DUXAP8 knockout inhibited tumor growth. Our study suggests that increased overexpression of pseudogene-derived LncRNA DUXAP8 is one of the important causes of non-small cell lung cancer. Thus, decreasing the expression level of LncRNA DUXAP8 will be a potential method for treatment of NSCLC, and detection of LncRNA DUXAP8 will be beneficial to NSCLC diagnosis and prognosis. Invasion and migration of tumor cells are one of the main reasons for the poor prognosis of NSCLC. LncRNA DUXAP8 knockout significantly inhibited cell invasion, whereas LncRNA DUXAP8 overexpression promoted cell invasion. LncRNA DUXAP8 could be a potential marker in tumor cell invasion of NSCLC. Ma *et al*. indicated that LncRNA DUXAP8 may epigenetically inhibit downstream target genes by binding to EZH2 and SUZ12 (two key components of PRC2) in gastric cancer, and further knockdown of DUXAP8 resulted in reduced EZH2 binding, SUZ12 binding, and H3K27me3 occupancy of the PLEKHO1 promoter[18]. In this study, we have not discussed how the LncRNA DUXAP8 mediated NSCLC progression, and further mechanical and detective studies should be required.

## 5 Conclusions

In conclusion, this study showed that LncRNA DUXAP8 was up-regulated in NSCLC tissues and cells and may be associated with the poor prognosis of patients with NSCLC. Various tumor stages and tumor sizes had different expression levels of LncRNA DUXAP8. LncRNA DUXAP8 overexpression significantly promoted the cells’ proliferation, enhanced invasion, and induced tumor growth. Conversely, LncRNA DUXAP8 knockout significantly suppressed cell proliferation, weakened invasion and inhibited tumor growth. The preliminary results imply that LncRNA DUXAP8 has potential as a promising diagnostic and predictive biomarker of NSCLC. However, the underlying mechanisms by which LncRNA DUXAP8 is involved in the regulatory functions of NSCLC cells remain to be comprehensively determined in the future.
